# Progress in Surgery

**Published:** 1896-05-16

**Authors:** 


### Progress in Surgery.

### BRAIN SURGERY.

**Brain Tumours.**—In an address delivered to the New York State Medical Society, Dr. M. Allen Starr[1] expresses his present opinion of the value of surgical measures in the treatment of brain tumours. He holds that only about seven per cent. of tumours of the brain are open to operation, and the opinions of other authorities recently expressed seem to confirm this. Discussing the question of the advisability of opening the skull for the relief of tension in cases of brain tumour where there is but little hope of being able to excise the growth, or even certainty of localisation, Starr on the whole tends to favour such operations. In this he agrees with Horsley, Park, and others, the first-named even going so far as to express his belief that the procedure may sometimes have a beneficial effect on the growth of the tumour. Starr thinks that the change in the circulatory conditions, consequent on the relief of tension, may in some cases aid in the effect of the absorptive power of some drugs. He states further that there are cases of brain tumour, not gummatous, in which the diagnosis is perfectly certain, which have recovered under the use of mercurial inunctions, together with very large doses of potassium iodide. Oppenheim,[2] in his recent monograph on brain tumours, emphasises this fact, and Starr reports a case in confirmation of it. The patient, a boy of sixteen, was perfectly free of syphilitic taint of any kind. He gradually developed all the symptoms associated with a tumour of the cerebellum pressing on the left side of the cerebral axis. Mercury and iodide were employed as a last resource, the iodide in very large doses, running up to 250 grains a day, with the result that improvement soon set in and became very marked, although some signs still remained. In three cases of cysto-sarcoma, marked improvement followed this line of treatment for a time at least. In a cerebellar case it failed.

As regards radical treatment the two essentials are (1) accurate localisation, and (2) the reasonable possibility of reaching the tumour. For purposes of localisation it is necessary to discriminate between the general symptoms common to all tumours—such as headache, optic neuritis, vomiting, vertigo, mental dulness, polyuria, &c.—and those which are strictly local in their significance, such as spasms, paralysis, aphasia, hemianopsia, cerebellar staggering, cranial nerve palsies, &c. Starr confirms Macewen's observation that on the side of a tumour or abscess there is sometimes found a clearer, higher-pitched, and more resonant note than on the opposite side. Horsley has recently pointed out that tenderness to pressure is often found over a tumour when tenderness to percussion cannot be elicited. It seems still impossible to distinguish between cortical and subcortical tumours. Another difficulty is that the nature of many tumours is impossible of determination till they are exposed, and large gliomata, which are quite unremovable, often give rise to comparatively few symptoms. It is only when the tumour is small, lies on but does not invade the cortex, and can be removed without serious damage, that the ultimate prognosis is absolutely good. A table of cases, gathered from medical literature up to January 1st, 1896, is given:—

Table of Results of Operations for Brain Tumour.

| | Cerebral. | Cerebellar. | Total. |
|---|---|---|---|
| Total number of cases operated upon for brain tumour ... ... ... ... ... | 137 | 25 | 162 |
| Cases in which tumour was not found ... | 39 | 9 | 48 |
| Cases in which tumour was found but not removed ... ... ... ... | 5 | 2 | 7 |
| Cases in which tumour was removed and patient recovered ... ... ... ... | 65 | 7 | 72 |
| Cases in which tumour was removed and patient died ... ... ... ... | 28 | 7 | 35 |

In analysing this table Starr refers to (1) the forty-eight cases in which the tumour was not found at operation. The error in diagnosis in most cases was due to the tumour causing symptoms referable to the motor cortex, while actually it was situated either deeply in the brain substance, or forward in the frontal region. It is well known that tumours so placed give rise to motor symptoms, which are quite undistinguishable from those of growths in the motor cortex. In another, but smaller, group, cerebellar tumour was diagnosed, although the growth was found in the frontal region. Numerous observers have met with cases in which ataxia of a cerebellar type was caused by frontal new growths. In a case recently reported by Stewart and Annandale[3] a cerebellar tumour was successfully diagnosed and removed, and the diagnosis was facilitated by correctly appraising the value of pain in the opposite frontal lobe as a sign of cerebellar tumour. (2) In seven cases a tumour was found, but proved to be impossible of removal. These were mostly large gliomata, which are always difficult to deal with. Early operation and careful selection of cases will diminish this class in future. (3) The third class includes cases in which the tumour was removed, of which there were 107, with 72 recoveries. It is gratifying to find that the proportion of recoveries from the operation, and also from the disease, has increased within recent years, and the writer is sure that the prognosis in such operations will continue to improve. The most satisfactory cases have been those in which the growth was in the motor, speech, or visual areas of the cortex.

A second table gives the tumours removed since 1893 and the results:—

Variety of Tumour Removed and Results—1893 to 1896.

| Variety. | Recovered. | Died. |
|---|---|---|
| Sarcoma ... ... ... ... ... ... | 10 | 5 |
| Glioma... ... ... .. ... ... | 3 | 1 |
| Glio-sarcoma ... ... ... ... ... | 2 | 0 |
| Cystic ... ... ... ... ... ... | 3 | 5 |
| Tubercular ... ... ... ... ... | 3 | 3 |
| Gumma ... ... ... ... ... | 1 | 2 |
| Fibroma ... ... ... ... ... | 0 | 1 |
| Angioma ... ... ... ... ... | 1 | 0 |
| Not stated ... ... ... ... ... | 3 | 4 |
| | 26 | 21 |

It will be seen that sarcomata are most common and give the best prognosis. Cysts are easily emptied, but the sacs are only removed with much damage to brain tissue. Gliomata also require wide incisions for their removal, but portions left can be taken away at subsequent operations with ultimately satisfactory results. When cerebral tuberculosis is the only evidence of the disease, operation is justifiable. In a few rare cases operation is advisable for gummatous tumours.

Stieglitz[4] reports three cases of operation for brain tumour with one recovery. The first case is interesting because a glio-sarcoma, the size of an almond, developed in the left motor area two years after the successful removal of a cystic tumour from the same region. It is urged that the simple evacuation of a cyst is not sufficient, but that the wall should, if possible, be excised also. This patient recovered. The fatal cases were examples of glio-sarcoma of the cerebellum and spindle-celled sarcoma of the motor cortex.

Some interesting remarks are made by Colman and Ballance[5] *apropos* of a case of sub-cortical cyst of the angular gyrus, causing aphasia, alexia, and agraphia, together with other symptoms characteristic of a cerebral tumour. Potassium iodide caused a temporary improvement, but to relieve inter-cranial tension a parallelogram of bone, three inches square, was removed from the skull. This was followed by immediate relief to the pain and sensory troubles, and the patient could read. A week later, as the pain, vomiting, and optic neuritis increased, the dura was opened and a cyst was exposed and evacuated. The fluid was ordinary plasma, which coagulated very readily. Great benefit followed this procedure for a time, but symptoms reappeared and the patient died. Post-mortem examination showed a large infiltrating glioma containing a cyst in the centrum ovale.

[1] Med. Record, Feb. 1, 1896. [2] "Die Geschwülste des Echirns," Wien, 1896. [3] Edin. Hosp. Reports. 1895. [4] Jour. Nerv. and Ment. Dis., Nov., 1895. [5] Lancet, March 21, 1896.

### DISEASE OF THE UPPER AIR PASSAGES.

(*Continued from page* 96.)

**Maxillary Sinus.**—Cyst of this sinus is discussed by C. H. Knight.[18] He recalls the usual division of cysts of the antrum into three varieties: (1) Those due to dilatation of a follicle in the mucous lining of the antrum; (2) those resulting from cystic degeneration of a polyp; and (3) dentigerous cysts, internal and external, the last developing outside the antrum and only reaching its interior by eroding and breaking down its bony wall. Dropsy of the antrum (*hydrops antri*), or an accumulation of non-purulent secretion within the cavity, due to obstruction of the *ostium maxillare*, is discarded by the majority of modern observers as a separate pathological condition. Thus Berger[19] has stated that nearly all cases called dropsy are really examples of dentary cyst, and that dropsy of the sinus without suppuration is so rare as to be almost unknown. Knight goes on to say that most of the cases reported as hydrops of the antrum are believed to be really cystic formations originating in the mucous membrane. The frequency of cysts of the antrum is not very great. Luschka (cited by Heath) found cystic growths in the antrum five times in sixty post-mortem examinations, but Beck (cited by Heath) found no instance of the kind in the antra of thirty subjects.

The diagnosis of these cysts is not always a simple matter. Heath cites Fergusson's case, in which preparations had been made to remove the upper jaw for a supposed solid tumour, which was shown to be fluid by a preliminary exploratory puncture; and refers to a case within his own knowledge, in which a very able surgeon removed the upper jaw before discovering the error of his diagnosis. A parallel case is reported by Marchant, in which a dentary cyst simulated a sarcoma, and was treated by resection of the superior maxilla; and Knight remarks that a similar error might readily have occurred in his own case but for exploration with the needle and trans-illumination, the wall of the tumour being so tense, and the mass itself so resistant, that many who examined it pronounced it a solid tumour. In the latter case it was impossible to distinguish a separate cyst wall. The author says it is difficult to believe that a cyst could have become so agglutinated to the walls of the antrum as to obliterate at all points a line of demarcation between its wall and the lining membrane of the antrum; its disappearance he explains by absorption, the cyst rupturing into the antral cavity, and every trace of the sac ultimately becoming effaced. The character of the fluid supported the cyst theory, for it resembled mucus in no respect, being a yellowish, slightly turbid serum. Cholesterin crystals were not found in this case.

**Antral Sinusitis and Empyema.**—Chiari[20] has recently related his experience in 58 cases of empyema, all recognised by perforation and exploratory washing. Nine times there existed in the alveolar process a fissure leading to the cavity. Rhinoscopic examination gives such certain diagnostic results that he has only fourteen times had recourse to exploratory puncture through the inferior meatus and irrigation to ensure a diagnosis. Irrigation of the maxillary sinus has rarely succeeded with him; he regards it as unsatisfactory for obtaining a cure, and it is impossible for the patient to practise for himself. He has perforated the alveolus eighteen times through the second bicuspid, fifteen times through the alveolus of the first molar, and four times through the second molar. Twelve patients were only observed a few days, and he could not report any more of them; but 27 were completely cured in from one to two weeks to four months, while the rest have been obliged to attend several months—even two years—and others have been only more or less improved. In the former cases the treatment consisted only of injections, irrigating and introducing iodoform gauze strips 70 centimetres long and 2 centimetres broad, which prevented decomposition and remained in the cavity eight to fifteen days. Lastly, the alveolar fistula was closed with iodoform gauze, and when suppuration ceased the fistula was kept closed for some time by a thin, hard caoutchouc canula applied against the gum. By shortening and diminishing the size of the canula he finally obtained closure of the fistula in its upper part. This method gave him the best and most rapid results. If cure was delayed too long he curetted the mucous lining through the alveolar opening. He never practised complete curetting of the mucous membrane in its whole extent. In three cases in which he perforated by the canine fossa by large openings, he twice obtained complete cure by iodoform gauze tamponning, and in one there was merely improvement. The operation through the canine fossa was always followed by great swelling of the cheek, and irrigations through the fistula in the canine fossa have always been much more painful than through the alveolar opening.

Hajek[21] also states that, reaching the sinus through the alveolar apophysis is, without doubt, the best and least disagreeable course, and that it is only in cases where the inferior part of the sinus is retracted (of which we are assured by the strong concavity of the external wall of the inferior nasal meatus) that it is necessary to avoid perforating the alveolar apophysis, because the trephine would almost infallibly enter the nasal cavity or the canine fossa, but not into the maxillary sinus. He cogently remarks that another point is to know if radical treatment through the canine fossa is indicated or not. The operation is more important, but success is uncertain. If the opening through the alveolar apophysis is not successful, that through the canine fossa, according to published cases, does not give encouraging results.

Two recently recorded cases are worthy of note. One was a *case of antral empyema in a child three weeks old*, and as it is generally supposed that maxillary sinusitis is not found before the seventh year, this case recorded by Rudaux[22] is very exceptional. Three weeks after birth the infant's eyes were red and œdematous; there was thrush on the mucous membrane of the mouth and gums, and over the canine fossa could be seen the premature eruption cf a tooth, which was quite loose. A few days afterwards pressure on the suborbital region led to discharge of the pus through the left nostril, and it was subsequently shown to come from the maxillary sinus. Pinder[23] records a case of antral empyema of seven years' duration, which perfectly recovered after removal of two molars, drilling of the antrum, and irrigations.

[18] N. Y. Med. Journ., Jan. 11, 1896. [19] Journ. of Surgery, vol. ii, 278, 1888. [20] Rep. of Vienna Laryng. Soc.; Journ of Surgery, Jan., 1896. [21] Ibid. [22] Journ. of Lar.. Feb., 1896; Ann. des Mal. de l'oreille, Sept., 1895. [23] Lancet, Oct. 18, 1895.

